# Symptoms and risk factors for hospitalization of COVID-19 presented in primary care: An exploratory retrospective study

**DOI:** 10.1007/s00508-021-01992-y

**Published:** 2022-02-11

**Authors:** Susanne Rabady, Kathryn Hoffmann, Markus Brose, Oliver Lammel, Stefanie Poggenburg, Monika Redlberger-Fritz, Karin Stiasny, Maria Wendler, Lukas Weseslindtner, Sonja Zehetmayer, Gustav Kamenski

**Affiliations:** 1 Department of General Health Studies, Division General and Family Medicine, Karl Landsteiner University of Health Sciences, Dr. Karl-Dorrek-Straße 30, 3500 Krems, Austria; 2 Unit Health Services Research and Telemedicine in Primary Care, Department of Preventive- and Social Medicine, Center for Public Health, Medical University of Vienna, Kinderspitalgasse 15, 1090 Vienna, Austria; 3 Praxis Dr. Oliver Lammel, Ramsau am Dachstein, Austria; 4 Ordination Dr. Stephanie Poggenburg, Hart bei Graz, Austria; 5 Center of Virology, Medical University Vienna, Vienna, Austria; 6 Center for Medical Statistics, Informatics and Intelligent Systems, Medical University of Vienna, Spitalg. 23, 1090 Vienna, Austria; 7 Karl Landsteiner Institute for Systematics in General Medicine, Angern, Austria

**Keywords:** Primary care, COVID-19, Predictors, Hospital admission

## Abstract

**Objective:**

To increase knowledge of discrete symptoms shall help to avoid misinterpretation of test results and to gain better understanding of associations between early symptoms and severe disease to provide additional criteria for targeted early interventions.

**Design:**

Retrospective observational study.

**Setting:**

Austrian GP practices in the year 2020, patients above 18 years were included.

**Participants:**

We recruited 25 practices which included 295 participants with a positive SARS-CoV‑2 test.

**Main outcome measures:**

Data collection comprised basic demographic data, risk factors and the recording of symptoms at several points in time in the course of the illness. Descriptive analyses for possible associations between demographics and symptoms were conducted by means of cross tabulation. Group differences (hospitalized yes/no) were assessed using Fisher’s exact test. The significance level was set to 0.05; due to the observational character of the study, no adjustment for multiplicity was performed.

**Results:**

Only one third of patients report symptoms generally understood to be typical for COVID‑19. Most patients presented with unspecific complaints. We found symptoms indicating complicated disease, depending on when they appear. The number of symptoms may be a predictor for the need of hospital care. More than 50% of patients still experience symptoms 14 days after onset.

**Conclusion:**

Unspecific symptoms are valuable indicators in the detection of early COVID‑19 disease that practitioners and the general public should be aware of also in the interpretation of low sensitivity tests. Monitoring patients using the indicators we identified may help to identify patients who are likely to profit from early intervention.

**Supplementary Information:**

The online version of this article (10.1007/s00508-021-01992-y) contains supplementary material, which is available to authorized users.

## Introduction

A central aspect in the containment of the coronavirus disease 2019 (COVID-19) pandemic is identification and isolation of possibly infectious persons, to prevent further spreading of the disease. Several studies were conducted with the goal of identifying diagnostic criteria that enable clinical differentiation between COVID-19 and non-COVID-19 infections: most investigations used data collected from hospitalized patients [1–6], i.e. from patients with severe disease. These studies have found high prevalence of fever (around 90%), dyspnea (up to 50%), cough (60–70%), and fatigue in patients with COVID-19. Several other studies evaluated self-reported data from symptom tracker apps or outpatient clinics [2, 7–10]. We could find only one investigation including additional data derived from primary care health records [9]. Studies conducted in non-hospitalized patients reported a lower prevalence of the symptoms mentioned above and a wide spectrum of additional symptoms, such as myalgia, rhinorrhea and/or nasal congestion, headache, sore throat, gastrointestinal and cardiovascular disturbances [7–9, 11]. Loss of taste and/or smell was found to be specific when present [8, 11]. All patients included in those studies had gone through a selection process before testing, by case definitions and testing criteria, by epidemiological factors or by previous investigations, such as computed tomography (CT) scans of the lungs. Some of these studies included only patients who had tested positive [10], others investigated patients who had tested positive or negative [8, 9]. Patients not fulfilling established criteria may have escaped testing, and the symptoms found may reflect testing criteria [7]. Some authors suspected that possibly a large proportion of COVID-19 cases are never tested and, thus, never recorded [12]. This has not been investigated so far.

### What this study can add

Patients presenting with other than the canonical symptoms might be overlooked by current testing strategies and screening tools. Patients with discrete and seemingly unsuspicious complaints tend to be mobile and can widely spread the disease. Awareness among stake holders as well as in the general public as to the wide range of uncharacteristic symptoms is most needed to promote low-threshold and high sensitivity testing, and to advise repeated testing if any symptoms are present, including unspecific, non-respiratory ones. This seems a requirement for effective containment strategies.

Austrian general practitioners (GPs) are entitled to make an individual testing decision according to clinical judgement like when there is no alternative explanation for the symptom presented. Austrian GP practices can send their own samples for severe acute respiratory syndrome coronavirus 2 (SARS-CoV-2) PCR to be analyzed via a surveillance network based at the Medical University Vienna, and from mid-October 2020 point of care testing for SARS-CoV‑2 in GP’s offices has become possible, thus 25% of our study practices were part of this network. GP practices in Austria are easy to access; in general, it is possible to walk in or to get an appointment on the same day.

Against this background, it was the aim of this study to assess early COVID-19 symptoms and their development in patients of different demographic groups in primary care as well as their possible associations with complications in the course of the disease.

## Patients, material and methods

This study was designed as an observational study in general practice in Austria. Recruitment of practices and participants took place between July 2020 and December 2020, thus comprising infections with the SARS-CoV‑2 wild type, which was the only one circulating in Austria at that time.

The Austrian Society of General Practice and Family Medicine invited their members, publicly funded GPs and their practices, to participate. For this purpose, first announcements and invitation letters were sent out between April and July 2020. After receiving a positive vote by the Ethics Committee of the Karl-Landsteiner University for Health Sciences, practices interested in participating were informed about the aims of this study in detail and after agreement study material was provided.

Participating GPs included patients above 18 years either after testing positive at the point of care or after reporting to their GP with a positive PCR test from another testing facility. These persons were invited to participate in this study. If they were willing to participate and after the provision of written informed consent, they were included in the study.

### Study material and data collection

A questionnaire using the open-source CDC program Epi Info 7 [13] was designed to record demographic and anamnestic data, comorbidities, medication groups and risk factors regarding COVID-19 (supplementary material 1). Data extracted from the electronic health records (EHR) of the practices were transferred to the questionnaire. Clinical parameters for assessing the patients’ health status over a period of 2 weeks starting with the day of symptom onset as day 1, were documented. Further assessment days were days 5, 7, 8, 10 and 14. Clinical data were self-reported in a monitoring sheet either by the patients themselves or acquired via telephone calls by the GPs. They were transferred to the questionnaire by the GP’s offices. Data regarding patients’ health status and symptoms were temperature >38°, blood pressure, heart rate, dyspnea, chest pressure, tightness of chest, malaise, weakness, headache, rhinitis, anosmia, ageusia, sore throat and gastrointestinal symptoms (supplemental material 2). These clinical parameters were selected using published studies of signs and symptoms of patients with COVID-19 [14, 15]. All data were pseudonymized before forwarding them to the study center where the data were checked for completeness and correct entries. Ambiguities were clarified by telephone call or e‑mail contact with the participating GP practice.

### Data analysis

In a first step we analyzed the data concerning demographics and symptoms during the acute phase of COVID-19 disease. These results are presented in this paper. Influence of comorbidities and other risk factors, e.g. patients’ age at hospitalization and long-term complications will be evaluated in a next step.

Data were converted into Excel files and accompanying statistical analysis was done using the statistical software program R (version 3.5.1) [16].

Participants’ demographics as well as symptoms were first analyzed descriptively. Descriptive analyses for possible associations between demographics and symptoms were conducted by means of cross tables. Group differences (hospitalized yes/no) were assessed using Fisher’s exact test. The significance level was set to 0.05; due to the observational character of the study, no adjustment for multiplicity was performed.

### Patient involvement

Study design included a protected patient information (PPI) leaflet. Patients were asked if they would like to participate in this study by their GPs, who informed them about the study’s goal and the proceedings involved. They were handed written information and were asked if they had any further questions. After giving written consent they either received a monitoring sheet to record their symptoms and to be returned after completion, or their GP arranged for regular monitoring telephone calls. The monitoring sheet also contained advice on situations requiring immediate medical attention, such as higher degree dyspnea. Patients remained under their physicians’ care during the full duration of the study, since it was the GPs who collected the data to pass them on to the study center after pseudonymization.

The GPs were asked to involve any patient with a SARS-CoV‑2 infection, they were instructed on how to collect patient data and how and why to perform the monitoring. They will be informed about the results of study and their possible consequences for patients’ care via newsletters, podcasts and publications.

## Results

Altogether, 25 GP practices and 295 patients from 7 of 9 Austrian federal states could be recruited. On average, the practices recruited 12 patients (SD 8.94, min 2–max 31).

As shown in Table 1 in detail, slightly more women than men were included in the study. In addition, the percentage of obese persons (body mass index [BMI] >30) was 19.0%, which is slightly higher than the general Austrian average of 16% [17]. On the other hand, only 7% of participants were smokers, which is less than half of the Austrian average of 20% [18]. Due to practical reasons smoking status was recorded following the GP’s reporting.

**Table 1:** Demographics (*N* = 295)

Variable	Subvariable	Percentage	*N*
Sex	Female	54.6	161
	Male	47.4	134
Age (years)	18–30	18.3	54
	31–50	34.2	101
	51–70	36.6	108
	71+	10.8	32
Obesity	Yes	19.0	56
	No	79.0	233
	NA	2.0	6
Smoking	Yes	6.8	20
	No	92.9	274
	NA	0.3	1

### Initial symptoms and development of symptoms

**Fig. 1:**
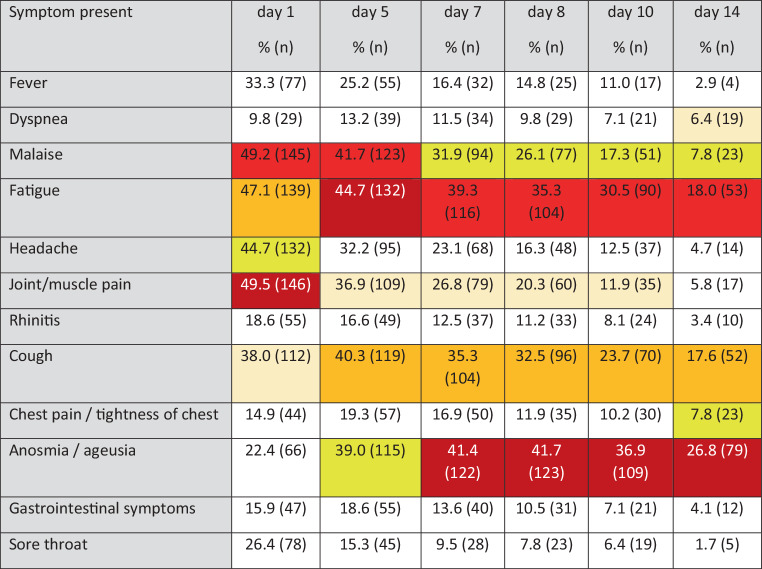
Symptoms reported on days 1, 5, 7, 8, 10, 14 (*N* = 295) (five most frequent symptoms color coded according to descending frequency: *dark red—red—dark yellow—yellow—light yellow*)

**Fig. 2:**
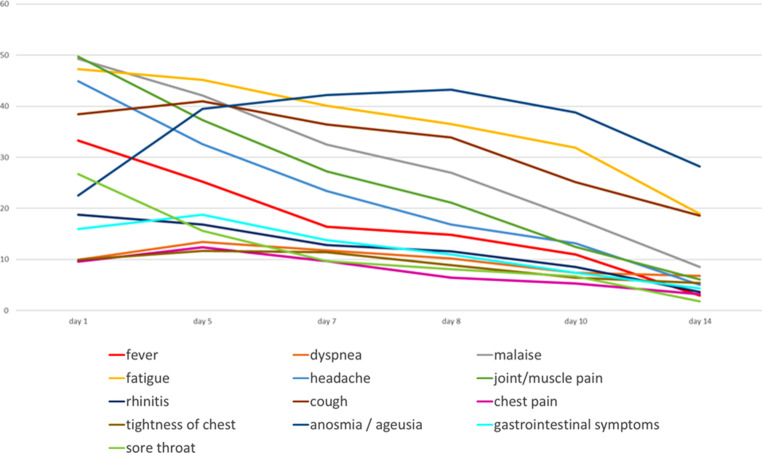
Frequencies of symptoms recorded on days 1–14

The most common out of the 13 symptoms to be selected were joint or muscle pain and malaise on day 1, each of them reported by half of the patients (Figs. 1 and 2). Loss of smell/taste was reported on day 1 by less than a quarter of patients but became the most frequently expressed complaint from day 7 onwards until the end of the observational period on day 14. Fatigue was the 3rd most prevalent symptom on day 1, and the most common symptom on day 5 and was still highly prevalent on day 10. Therefore, fatigue was found to be the most persistent of symptoms of all. Cough turned out to be a less common symptom on day 1 (5th of 13 symptoms), becoming more frequent from day 5 onwards. Fever >38° was reported by one third of participants as an initial symptom.

### Associations between symptoms and hospitalization

**Fig. 3:**
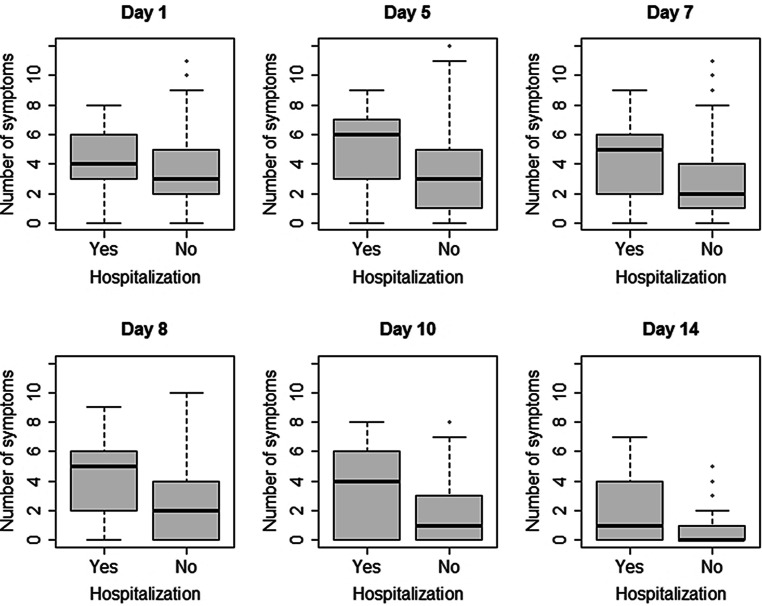
Number of symptoms in comparison of hospitalized to non-hospitalized patients

Analysis for associations of symptoms with the likelihood of hospitalization yielded the following results: for presence of fever and/or malaise from day 5 onwards we found significant associations with the need for hospitalization sometime in the further course of the disease. Headache showed a significant association if present on day 10. The likelihood for hospitalization was significantly increased in patients with either dyspnea, fatigue, tightness of chest and cough. In contrast, persons with rhinitis, sore throat, chest pain and anosmia as an initial symptom were less likely to need hospital care (Table 2; Fig. 3).

**Table 2:** Hospital admission vs. non-admission in relation to the presence of a symptom

	Day 1: *n* (%)			Day 5: *n* (%)			Day 8: *n* (%)			Day 10: *n* (%)		
Symptom present	No Hosp.	Hosp.	*p*	No Hosp.	Hosp.	*p*	No Hosp.	Hosp.	*P*	No Hosp.	Hosp.	*P*
Fever	68 (32.1)	9 (47.4)	0.271	43 (21.5)	12 (66.7)	<0.001	17 (11.1)	8 (50.0)	<0.001	10 (7.1)	7 (50.0)	<0.001
Dyspnea	23 (8.6)	6 (25.0)	0.026	33 (12.3)	6 (27.3)	0.099	24 (9.2)	5 (23.8)	0.050	16 (6.1)	5 (23.8)	0.013
Malaise	130 (48.1)	15 (62.5)	0.257	106 (39.3)	17 (77.3)	0.001	61 (23.2)	16 (72.7)	<0.001	41 (15.7)	10 (47.6)	0.001
Fatigue	123 (45.6)	16 (66.7)	0.076	116 (43.0)	16 (72.7)	0.013	89 (33.8)	15 (68.2)	0.003	77 (29.5)	13 (61.9)	0.005
Headache	123 (45.6)	9 (37.5)	0.585	85 (31.6)	10 (45.5)	0.273	42 (16.0)	6 (27.3)	0.287	29 (11.2)	8 (38.1)	0.001
Joint/muscle pain	134 (49.6)	12 (50.0)	1.000	97 (35.9)	12 (54.5)	0.132	52 (19.8)	8 (36.4)	0.118	29 (11.2)	6 (28.6)	0.048
Rhinitis	51 (19.0)	4 (16.7)	1.000	45 (16.7)	4 (18.2)	0.772	28 (10.6)	5 (22.7)	0.154	21 (8.0)	3 (14.3)	0.403
Cough	97 (36.2)	15 (62.5)	0.020	104 (38.8)	15 (68.2)	0.014	82 (31.4)	14 (63.6)	0.005	60 (23.3)	10 (47.6)	0.027
Chest pain	27 (10.0)	1 (4.2)	0.713	35 (13.1)	1 (4.5)	0.332	16 (6.1)	2 (9.1)	0.639	15 (5.7)	0 (0.0)	0.613
Tightness of chest	24 (8.9)	5 (20.8)	0.073	29 (10.8)	5 (22.7)	0.156	22 (8.5)	3 (13.6)	0.427	14 (5.4)	4 (19.0)	0.035
Anosmia/ageusia	61 (22.7)	5 (20.8)	1.000	102 (37.9)	13 (59.1)	0.084	110 (41.8)	13 (59.1)	0.178	98 (37.7)	11 (52.4)	0.273
Gastrointest. symptoms	43 (15.9)	4 (16.7)	1.000	49 (18.1)	6 (27.3)	0.442	28 (10.7)	3 (13.6)	0.719	18 (6.9)	3 (14.3)	0.197
Sore throat	74 (27.6)	4 (16.7)	0.337	41 (15.3)	4 (20.0)	0.530	17 (6.5)	6 (27.3)	0.002	12 (4.6)	7 (33.3)	<0.001

**Fig. 4:**
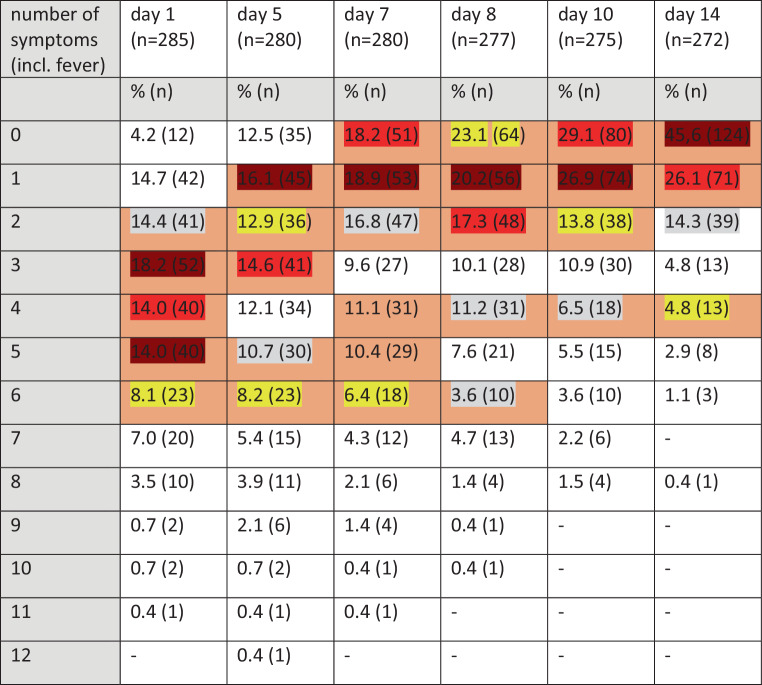
Number of symptoms by days 1, 5, 7, 8, 10, 14 (color coding for the five most frequent symptoms on a given measuring point (days 1, 5, 7 ,8, 10, 14) according to descending frequency: *dark red—red—dark yellow—yellow—grey*)

COVID-19 seems to start with several symptoms simultaneously: Nearly half of the participants reported 3–5 symptoms on day 1, 10 participants had not reported symptoms on day 1, with 3 of them having become symptomatic by day 5 and 7 remained asymptomatic (Fig. 4).

Of the patients 68% reported symptoms persisting on day 10, and half the sample still had complaints on day 14.

A higher number of symptoms was associated with higher probability of hospital admission. This was significant for days 7 and 8 (Fig. 3).

## Discussion

### Initial symptoms and development of symptoms

We found early symptoms to be mostly unspecific and often discreet, with joint or muscle pain, malaise and fatigue being the most common symptoms (Figs. 1 and 2). One of our findings is that none of the 13 symptoms in our selection is either sensitive or specific enough for the early stage of COVID-19 to serve as testing criteria, which is supported by several of the more recent studies based at least partly on data from primary care [7, 9].

Symptoms generally considered to increase the likelihood for COVID-19 diagnosis, i.e. fever, dyspnea and loss of taste and/or smell [7, 9, 10, 19, 20] were found to be comparatively rare, at least at early stages of the disease (Figs. 1 and 2). On day 1 less than 30% of patients reported fever, less than one third (25.9%) reported loss of smell or taste and only 13.6% suffered from dyspnea, which renders those symptoms not well suited as testing criteria. These results differ from other studies that had derived data from hospitalized patients exclusively, or in combination with self-reported data using symptom apps. All of them were conducted in patients who had been tested according to pre-established testing criteria [8, 10]. Our findings suggest that neither presence nor absence of any symptom is suitable to rule out COVID-19, and confirmed results from another study showing that patients reporting any kind of symptoms of a broad range of diseases profit from testing without delay and without restrictions [21].

Fever, cough, and dyspnea have been considered relevant for case definitions and used as screening criteria in many countries for a long time, and to this day are widely understood as characteristic by the general public and mass media. According to our findings this could be an obstacle to correct early diagnosis and case finding. In our sample, none of those symptoms was experienced by more than slightly over a third of the patients at any time during the illness.

This finding may have some impact on testing strategies. Austria like other countries propagates low threshold testing by self-testing kits, which mostly lack external validation of their sensitivity and specificity [22]. Sensitivity varies considerably altogether between the lateral flow tests (LFT) being marketed at the time of the study [23]. We found presence of any of the discreet and unspecific symptoms we investigated to possibly indicate COVID‑19 infection and to increase pre-test probability, thus further reducing sensitivity. Users and decision makers need to be aware of this to avoid misinterpretation of a negative test result [24].

Anosmia is known to be the most specific symptom [8] but according to our results tends to appear in the later course of the disease. As an initial symptom we could trace it in only 22% of patients, but in twice as many on days 7 and 8 (Fig. 1). Menni [8] identified this symptom in 64.5% of patients and concluded that this symptom could help early diagnosis. Our findings do not support this conclusion. The difference may be caused by the time of detection or selection bias and thus shows the relevance of early investigation of symptoms at a low threshold point of care [14].

This finding should lead to reconsider contact-tracing strategies: finding the symptom anosmia might indicate delayed diagnosis and should prompt an extension of the contact tracing period to at least 7 days before the infection was detected.

### Associations between symptoms and hospitalization

We found several associations between symptoms and the need for hospitalization. Our findings suggest that dyspnea is prone to lead to admission to hospital when present on day 1, as well as on day 10 and significantly on day 14. Fatigue as well as malaise through the whole course of the disease starting from day 5 and cough persisting on day 8 seem to be associated with higher rates of hospitalization, fever only if present from day 5 on (Table 2; Fig. 3). Dyspnea has been shown to be predictive for hospital admission in one systematic review and a meta-analysis [2, 19], which is in line with our findings. No other predictive symptoms has consistently been identified so far. Our findings on persisting symptoms at the end of the observational period fit in well with recent studies on Long Covid [25, 26].

We observed a higher number of symptoms (3 or more) on days 7 and 8 to be associated with a significantly higher probability of hospital admissions (Fig. 3). Patients in the non-hospitalized group experience a peak in number of symptoms on day 1 to day 5. Patients in the hospitalized group had more symptoms from the start and experienced a further rise on day 5 as well as a markedly slower decrease. This could indicate that patients might profit from being closely monitored: Finding a rise in number of symptoms should arouse suspicion of an imminent severe course of disease. This might, if corroborated, even allow identification of individual cut-off points to introduce innovative early interventions presently under discussion like budesonide, low-molecular heparins or monoclonal antibody therapy [27, 28].

Over 50% of patients reported symptoms at the end of the observational period on day 14, and more than two thirds on day 10, when the isolation period usually ends. Mostly this concerns loss of taste or smell or fatigue. This coincides with the most common complaints reported by patients suffering from Long Covid [25]. Regarding the fact that Long Covid is in many cases not a trivial complaint but may lead to delayed and severe complications [29], it seems justifiable to recommend medical examination after isolation to decide if and when a patient can be considered healthy and safe to return to physical activity and/or work.

### Strengths and limitations of this study

This study to our knowledge is the first one to investigate data on the course of COVID-19 collected exclusively from patients in primary care. The GPs were free to make their own testing decision according to their clinical judgement, and they followed each patient individually from day 1 to day 10 or 14 in most cases. Only 10% of the patients included in our study approached primary care after having been tested according to formal testing criteria by another testing facility. Most other studies [2, 7, 9, 10] recruited patients mainly or exclusively via symptom apps or in hospital care, after the testing decision had been made according to established testing criteria, which makes them less likely to detect early symptoms not already known to be associated with COVID-19.

The study has several limitations though. We could recruit 25 practices in 7 out of the 9 Austrian provinces. The average number of patients included per practice was 12 (SD 8.9). The limited number of patients is probably due to the increased workload under difficult working conditions during the pandemic in combination with a rather extensive questionnaire and the need to follow patients over a longer period as well as the effort not being remunerated. We have to reckon with possible recruiting bias; however, the number of cases needed to identify group differences was calculated in advance, and this number has been reached. Our overall results are in accordance with our preliminary result analyses.

Another limitation is that not all data on symptoms were provided by the GPs, particularly on temperature. Most likely this applies mainly for symptoms which were not present but this will have to be clarified by further research. Some patients (approximately 10%) were not diagnosed in primary care, so a possible confounder testing criteria cannot entirely be ruled out. We are likely to have overestimated some symptoms on day 1, because a proportion of patients may have unknowingly been diagnosed later than that.

We could not detect specific patterns of symptom combinations. This may be due to the limited sample size.

## Conclusion

We could demonstrate a variety of unspecific symptoms to be clearly more common than those widely understood to be typical of COVID-19. Understanding this can help to avoid missing infectious patients because inappropriate testing methods like testing kits of low sensitivity are being used. This applies particularly since negative antigen tests are accepted for the Green Pass, and new waves of infections with new variants are considered a threat to re-opening strategies after lock-down measures. Negative results in persons with any of the symptoms we identified should be confirmed by PCR testing.

We found several symptoms possibly indicating future complications. This knowledge in conjunction with timely identification in primary care could help to avert severe disease in some cases. To facilitate follow-up in primary care, patients need to be either diagnosed there, or to reliably report to their GP if tested positive.

### Implications for further research

Data collected in primary care settings can provide additional information and can offer a wider spectrum of understanding COVID-19 disease. The results of our exploratory retrospective study should be controlled by a prospective study.

We did not record data on patients who tested negative or were not tested at all. Future investigations into this topic should aim at recording symptoms in all patients reporting for suspected SARS-CoV‑2 infection.

## Supplementary Information

Supplemental Material 1 Questionnaire

Supplemental Material 2 Monitoring sheet
