# Supplementary material for: Symptoms and risk factors for hospitalization of COVID-19 presented in primary care: An exploratory retrospective study
Source: Wien Klin Wochenschr. 2022 Feb 11;134(9-10):335–43. doi: 10.1007/s00508-021-01992-y (PMC8852901; doi:10.1007/s00508-021-01992-y)
Supplement: Supplementary file 1 — Supplemental Material 1 Questionnaire [file 508_2021_1992_MOESM1_ESM.pdf]

PrimeRisk C-19 Fälle in häuslicher Betreuung S1

ID Nr.Praxis | ID Nr. Patient | Sex m=0,w=1 | Alter | Symptombeginn am | PCR Test am | PCR positiv

egativ PCR Test bei Verdacht Covid19 | Nur klin.Symptome | Angehoeriger pos getestet | Sonstiger Kontakt pos

Risikofaktoren BMI | Rauchen | Influenzaimpfung | Pneumokokkenimpfung

Komorbiditäten Asthma bronchiale | COPD | Diabetes | KHK | Kard.Insuff. | Hypertonie | Kardiovaskul.KH Sonstige

Renale Insuffiz. | Lebererkrankung | Aktive Tumorerkrank | TBVT/Pulmonalembol | Zerebrovask. Erkrank | Demenz | Psych. Erkrank

Dauer-Medikamente Antihypertensiva | ACE-Hemmer,ARB | COPD-Medikation | Immunsuppressiva-Kortison | Metformin

Antidiabetika-orale Kombination | Insulin | Psychoaktive Medikation | Orale Antikoagulation | Niedermolekulares Heparin

Symptome Tag 1: T C° | HF durchschn. | Dyspnoe ausgeprägt | KH Gefühl stark | Schwaeche ausgepraegt | Kopfschmerz

Gliederschmerzen | Rhinitis | Husten | Thoraxdruck/schmerz | Thorakale Beklemmung | Anosmie,Agustie

Gastrointest.Sympt. | RR | Halsschmerzen | Anderer Tag Symptomerfassung

Symptome Tag 5: T C° | HF durchschn. | Dyspnoe ausgeprägt | KH Gefühl stark | Schwaeche ausgepraegt | Kopfschmerz

Gliederschmerzen | Rhinitis | Husten | Thoraxdruck/schmerz | Thorakale Beklemmung | Anosmie,Agustie

Gastrointest.Sympt. | RR | Halsschmerzen | Anderer Tag Symptomerfassung

Symptome Tag 7: T C° | HF durchschn. | Dyspnoe ausgeprägt | KH Gefühl stark | Schwaeche ausgepraegt | Kopfschmerz

Gliederschmerzen | Rhinitis | Husten | Thoraxdruck/schmerz | Thorakale Beklemmung | Anosmie,Agustie

Gastrointest.Sympt. | RR | Halsschmerzen | Anderer Tag Symptomerfassung

Symptome Tag 8: T C° | HF durchschn. | Dyspnoe ausgeprägt | KH Gefühl stark | Schwaeche ausgepraegt | Kopfschmerz

Gliederschmerzen | Rhinitis | Husten | Thoraxdruck/schmerz | Thorakale Beklemmung | Anosmie,Agustie

Gastrointest.Sympt. | RR | Halsschmerzen | Anderer Tag Symptomerfassung

ID Nr.Praxis ID Nr. Patient

PrimeRisk C-19 Fälle in häuslicher Betreuung S2

Symptome Tag 10: T C° HF durchschn. Dyspnoe ausgeprägt KH Gefühl stark Schwaeche ausgepraegt Kopfschmerz

Gliederschmerzen Rhinitis Husten Thoraxdruck/schmerz Thorakale Beklemmung Anosmie,Agustie

Gastrointest.Sympt. RR Halsschmerzen Anderer Tag Symptomerfassung

Symptome Tag 14+: T C° HF durchschn. Dyspnoe ausgeprägt KH Gefühl stark Schwaeche ausgepraegt Kopfschmerz

Gliederschmerzen Rhinitis Husten Thoraxdruck/schmerz Thorakale Beklemmung Anosmie,Agustie

Gastrointest.Sympt. RR Halsschmerzen Anderer Tag Symptomerfassung

Fiebertage>38° Woche1 Fiebertage>38° Woche2 Anzahl Covid19 Erkrankter Umfeld

KH-Aufnahme noetig wegen: Nierenversagen Kreislaufversagen Fieberhöhe/Dauer Oedeme Dyspnoe schwer

KH Aufnahme am wegen sonstiger Ursache (Freitext) Geplantes Ende Monitoring am

Abbruch häusliche Betreuung durch Arzt Abbruch häusliche Betreuung durch Patient

Komplikationen während der Erkrankung Thrombose Pulmonalembolie MCI Stroke

Andere Komplikationen während der Erkrankung. Freitext NMH s.c.während Erkrankung

Längerfristige Komplikationen (innerhalb der ersten 3 Monate) Thrombose Pulmonalembolie MCI Stroke Lungenfibrose

Längerfristige andere Komplikationen (innerhalb der ersten 3 Monate) Freitext

Tod am **Todesursache**

AK-Nachtestung geplant Datum Nachtestung Titerhoehe
