# Supplementary material for: Symptoms and risk factors for hospitalization of COVID-19 presented in primary care: An exploratory retrospective study
Source: Wien Klin Wochenschr. 2022 Feb 11;134(9-10):335–43. doi: 10.1007/s00508-021-01992-y (PMC8852901; doi:10.1007/s00508-021-01992-y)
Supplement: Supplementary file 2 — Supplemental Material 2 Monitoring sheet [file 508_2021_1992_MOESM2_ESM.pdf]

Bogen zur Selbstbeobachtung Covid 19 Name:____ Datum Testung:______________________

Wenn Sie bei <u>Atemnot</u> einen Wert über 3 eintragen, oder sich plötzlich schlecht fühlen, rufen Sie bitte sofort Hausarzt/Hausärztin, oder 144 an.

Datum	Temp. höchste / Tag	Krankheits-gefühl	Atemnot	Schwäche	Schmerzen		Husten	Brust-enge	Weitere Symptome	
		0 1 2 3 4 5 6 7 8 9 10	0 1 2 3 4 5 6 7 8 9 10	0 1 2 3 4 5 6 7 8 9 10	Glieder	Ja/nein	Ja/nein	Ja/nein	Geschmack	Ja/nein
					Kopf	Ja/nein			Geruch	Ja/nein
					Hals	Ja/nein			Durchfall	Ja/nein
					Ohren	Ja/nein			Bauchweh	Ja/nein
		0 1 2 3 4 5 6 7 8 9 10	0 1 2 3 4 5 6 7 8 9 10	0 1 2 3 4 5 6 7 8 9 10	Glieder	Ja/nein	Ja/nein	Ja/nein	Geschmack	Ja/nein
					Kopf	Ja/nein			Geruch	Ja/nein
					Hals	Ja/nein			Durchfall	Ja/nein
					Ohren	Ja/nein			Bauchweh	Ja/nein
		0 1 2 3 4 5 6 7 8 9 10	0 1 2 3 4 5 6 7 8 9 10	0 1 2 3 4 5 6 7 8 9 10	Glieder	Ja/nein	Ja/nein	Ja/nein	Geschmack	Ja/nein
					Kopf	Ja/nein			Geruch	Ja/nein
					Hals	Ja/nein			Durchfall	Ja/nein
					Ohren	Ja/nein			Bauchweh	Ja/nein
		0 1 2 3 4 5 6 7 8 9 10	0 1 2 3 4 5 6 7 8 9 10	0 1 2 3 4 5 6 7 8 9 10	Glieder	Ja/nein	Ja/nein	Ja/nein	Geschmack	Ja/nein
					Kopf	Ja/nein			Geruch	Ja/nein
					Hals	Ja/nein			Durchfall	Ja/nein
					Ohren	Ja/nein			Bauchweh	Ja/nein

Bogen zur Selbstbeobachtung Covid 19 Name:____ Datum Testung:______________________

Wenn Sie bei <u>Atemnot</u> einen Wert über 3 eintragen, oder sich plötzlich schlecht fühlen, rufen Sie bitte sofort Hausarzt/Hausärztin, oder 144 an.

Datum	Temp. höchste / Tag	Krankheits-gefühl	Atemnot	Schwäche	Schmerzen		Husten	Brust-enge	Weitere Symptome	
		0 1 2 3 4 5 6 7 8 9 10	0 1 2 3 4 5 6 7 8 9 10	0 1 2 3 4 5 6 7 8 9 10	Glieder	Ja/nein	Ja/nein	Ja/nein	Geschmack	Ja/nein
					Kopf	Ja/nein			Geruch	Ja/nein
					Hals	Ja/nein			Durchfall	Ja/nein
					Ohren	Ja/nein			Bauchweh	Ja/nein
		0 1 2 3 4 5 6 7 8 9 10	0 1 2 3 4 5 6 7 8 9 10	0 1 2 3 4 5 6 7 8 9 10	Glieder	Ja/nein	Ja/nein	Ja/nein	Geschmack	Ja/nein
					Kopf	Ja/nein			Geruch	Ja/nein
					Hals	Ja/nein			Durchfall	Ja/nein
					Ohren	Ja/nein			Bauchweh	Ja/nein
		0 1 2 3 4 5 6 7 8 9 10	0 1 2 3 4 5 6 7 8 9 10	0 1 2 3 4 5 6 7 8 9 10	Glieder	Ja/nein	Ja/nein	Ja/nein	Geschmack	Ja/nein
					Kopf	Ja/nein			Geruch	Ja/nein
					Hals	Ja/nein			Durchfall	Ja/nein
					Ohren	Ja/nein			Bauchweh	Ja/nein
		0 1 2 3 4 5 6 7 8 9 10	0 1 2 3 4 5 6 7 8 9 10	0 1 2 3 4 5 6 7 8 9 10	Glieder	Ja/nein	Ja/nein	Ja/nein	Geschmack	Ja/nein
					Kopf	Ja/nein			Geruch	Ja/nein
					Hals	Ja/nein			Durchfall	Ja/nein
					Ohren	Ja/nein			Bauchweh	Ja/nein
